# Validation of tool to assess pediatric residents’ knowledge of development and behavior

**DOI:** 10.1590/1984-0462/2023/41/2021372

**Published:** 2023-01-20

**Authors:** Mariana Grando Pegoraro, Eduardo Juan Troster

**Affiliations:** a Hospital Israelita Albert Einstein, São Paulo, SP, Brazil.

**Keywords:** Child development, Child behavior, Education, medical, Knowledge, Clinical competence, Validation study, Desenvolvimento infantil, Comportamento infantil, Educação médica, Conhecimento, Competência clínica, Estudo de validação

## Abstract

**Objective:**

This study aimed to create and validate an instrument to measure pediatric residents’ knowledge about development and behavior.

**Methods:**

This was a longitudinal study with the consecutive application of questionnaires to validate an instrument of analysis. The modified Delphi technique was used for validation, which involved judges who were selected based on their expertise. Judges, who were renowned for their knowledge of the subject and willing to participate, were chosen from different states of Brazil. A convenience sample was obtained. The original questionnaire included 45 open questions divided into 13 relevant thematic axes on development and behavior.

**Results:**

After the third round using the Delphi technique, the whole questionnaire had a validity index of more than 80% on scope and relevance as well as all thematic axes, and the 44 final questions.

**Conclusions:**

The whole questionnaire was considered validated by the 14 expert judges who participated in the study.

## RESUMO

**Objetivo:**

Construir e validar o conteúdo de um instrumento de análise do conhecimento acerca do diagnóstico de transtornos de desenvolvimento e comportamento entre residentes de pediatria.

**Métodos:**

Foi realizada uma aplicação consecutiva de questionários, visando à validação de um instrumento de análise. A metodologia utilizada para a validação foi a técnica Delphi modificada. Juízes especialistas procedentes de diferentes Estados do Brasil foram selecionados com base em sua expertise no tema, por meio de uma amostra de conveniência. O primeiro questionário submetido continha originalmente 45 questões de múltipla escolha, divididas em 13 eixos temáticos relevantes, sobre desenvolvimento e comportamento.

**Resultados:**

Após a terceira rodada da metodologia, o questionário como um todo obteve mais de 80% de índice de validade de conteúdo sobre abrangência e relevância, assim como todos os eixos temáticos e as 44 questões finais.

**Conclusões:**

O questionário como um todo foi considerado validado pelos 14 juízes especialistas que participaram do estudo.

## INTRODUCTION

The first 5 years of a child's life is the period when the developing brain is most susceptible to stimulation.^1^ The cognitive and socioemotional skills developed during this time will impact academic achievement, health, and general well-being in adulthood.^2,3^

Despite affecting millions of children worldwide, developmental disorders are under diagnosed and there is a significant lack of available studies, especially in developing countries.^3,4^

According to studies conducted in the United States, it is estimated that at least one in five children present with either developmental and/or behavioral disorders.^5,6^ Despite the significant number of cases, most children remain undiagnosed and, subsequently, untreated. Pediatricians identify only 50% of cases before children begin preschool.^7^

Early diagnosis is important for timely intervention, with better long-term outcomes. The child's general pediatrician should be the professional responsible for surveillance.^5,8,9^

In Brazil, the National Board of Medical Residency requires the study of this subject in its accredited programs but does not stipulate minimum credit requirements.^10^ For a subject so prevalent in the pediatric environment, and with such significant impact on individual and collective health, the emphasis given in our pediatric medical residency programs is low. In contrast, one of the concerns of medical educators is guaranteeing the teaching quality of the residents. One of the ways of reaching this is using competency assessment methods.^11^

Considering that the pediatrician is the professional who would diagnose these disorders, we justified the elaboration of this project. We hypothesized that a validated questionnaire would successfully measure pediatric residents’ knowledge and ability to diagnose developmental and behavioral disorders. Our theoretical framework is that a questionnaire could identify knowledge gaps in residents. Thus, we aimed to build and validate an instrument to assess the knowledge regarding the diagnosis of behavior and development disorders.

## METHOD

We used a consecutive application of questions to validate an instrument of analysis based on validation techniques. The method used to consult specialists was the modified Delphi technique.

We obtained validation by submitting questionnaires to a group of judges:

1. Physicians with medical residency in pediatrics or child neurology and/or with a title of specialist in child neurology;
2. With at least 3 years of working experience in the field of pediatric development.

Judges, who were renowned for their knowledge of the subject and willing to participate, were chosen from different states of Brazil. We used a convenience sample, predominantly using specialists recommended by the Brazilian Society of Child Neurology, prioritizing those with the most titles. We stipulated a minimum of 10 judges for the first round of this study, according to the sample size calculation to obtain validity.

We sent each selected judge an electronic invitation stating the project's objectives and an Informed Consent Form. Those judges who agreed to participate in the study received the instrument via Google Forms, an online data collection tool.

Judges received questionnaires containing:

1. Expert/Specialist Characterization Data and
2. 45 multiple choice questions, each with 4 multiple choice alternatives in which only one option was correct, distributed across 13 thematic axes.

We used a 4-point Likert scale, ranging from 1 (totally disagree) to 4 (totally agree), in order to gauge the opinion of the experts. We evaluated questions, thematic axes, and the questionnaire as a whole.

The initial questionnaire, containing 45 questions, with 4 multiple choice alternatives each, and distributed across 13 thematic axes, was elaborated with emphasis on diagnosis. We based this questionnaire on the areas of greatest importance within the subject, using two main bibliographic references (i.e., one American and one Brazilian),^9,11,12^ in addition to important scientific articles.^8,13–18^ The questionnaire is available by request with the corresponding author.

The preparation of the questionnaire followed guideline recommendations for the preparation of multiple choice tests.^19^

A single researcher elaborated the questions using the aforementioned bibliography and taking into account all the cares as listed above. The researcher responsible for the idealization and construction of the questions had a degree in general pediatrics.

The 13 thematic axes were chosen based on relevance within the subject in question and literature thereof, and are as follows:

1. Screening and surveillance of developmental and behavioral disorders, recognition of normal development, and warning signs regarding developmental delays;
2. Intellectual disability;
3. Specific learning disorders, including major differential diagnoses such as dyslexia, dyscalculia, and dysgraphia;
4. Secondary learning disorders due to visual and auditory difficulties, organic diseases, and/or abuse and ill treatment;
5. Follow-up of high-risk newborns;
6. Excessive crying of infants;
7. Sleep disorders;
8. Speech and language disorders;
9. Eating disorders;
10. Anxiety and depression;
11. Temperament and disruptive behavior;
12. Autism spectrum disorders; and
13. Attention deficit hyperactivity disorder.

We measured the Content Validity Index (CVI) and we expected to obtain a CVI greater than 0.8 (or 80%) to finish the current round of the Delphi technique and thus consider the questionnaire validated.

We evaluated the agreement between the judges using a coefficient of agreement appropriate to the distribution of responses, such as Gwet's AC2,^20^ with ordinal weight, and the coefficients were accompanied by intervals of 95% confidence and p-values. We compared the coefficients of agreement to the classification present in Altman,^21^ which considers the following: “poor” coefficients lower than 0.2, “reasonable” those between 0.2 and 0.4, “moderate” those between 0.4 and 0.6, “good” between 0.6 and 0.8, and “excellent” those above 0.8.

The Research Ethics Board of the Hospital Israelita Albert Einstein approved this study under the registration number 2.955.041 dated October 10, 2018.

## RESULTS

We invited a total of 37 specialists to participate in the project. Of these, 17 declined participation, and 6 did not respond to the invitation.

A total of 14 specialists, aged 31–66 years, equally divided between men and women, participated in the study – the mean time since their graduation was 22 years, ranging from 6 to 43 years. The majority of specialists (10/14) practiced child neurology. Of the 14 participants, 6 had at least 15 years of working experience in the field of study, and 9 had at least a master's degree. Table 1 provides the characteristics of the experts.

**Table 1:** Experts’ characterization data.

Data	Description	Value
Age	Mean (DP)	47 (12)
	Minimum and maximum	31–66
Gender	Male	7 (50%)
	Female	7 (50%)
Area of activity	Child neurology developmental	10 (71%)
	Behavioral pediatrics	4 (29%)
Time of experience in the area of activity, years	3–5	4 (19%)
	5–10	2 (14%)
	10–15	2 (14%)
	>15	6 (43%)
Professional degree	Post-doctoral	1 (7%)
	Doctorate degree	3 (21%)
	Master's degree	5 (36%)
	Post-graduation	2 (15%)
	Residency	3 (21%)
Type of institution	Public	1 (7%)
	Private	4 (29%)
	Philanthropic	1 (7%)
	Public + private	6 (43%)
	Public + private + philanthropic	2 (14%)

Of the 45 questions submitted in the first round, 17 presented a CVI below 0.8 requiring reformulation. Of the 13 axes, 4 did not obtain satisfactory CVI for relevance and/or comprehensiveness, and the questionnaire, as a whole, did not present sufficient relevance. Table 2 provides CVIs for axes and the questionnaire obtained in the first round.

**Table 2:** Analysis of Content Validity Index for axes and the questionnaire as a whole obtained in the first round.

Axes	CVI relevance %	CVI scope %
Axis 1	100	71.4
Axis 2	92.9	78.6
Axis 3	92.9	85.7
Axis 4	100	92.9
Axis 5	92.9	92.9
Axis 6	100	71.4
Axis 7	92.9	85.4
Axis 8	78.6	78.6
Axis 9	92.9	85.7
Axis 10	100	100
Axis 11	100	100
Axis 12	100	100
Axis 13	92.9	92.9
Questionnaire	78.6	85.7
Total	93.9	87.2

CVI: Content Validity Index. Measurement of the CVI in percentage for axes. The axes with CVI lower than 0.8 for relevance and/or scope are highlighted.

After the statistical analysis of the 17 questions, we eliminated 6 with CVI below 0.8 due to structural errors, reformulated 11, and inserted 6 new questions. In addition, the 20 questions that obtained satisfactory CVI in the first round underwent minor changes, as suggested in the open field by the experts.

In the second round, 10 of the original 14 specialists participated in the evaluation of the instrument. We evaluated a total of 31 question reformulations, in addition to the 6 new questions, the scope of axes 1, 2, and 6, the relevance and scope of axis 8, and the questionnaire as a whole.

Only three questions did not obtain the necessary CVI after the second round. After the modifications, axes 1, 2, 6, and 8 presented sufficient coverage, and axis 8 also showed sufficient relevance. Regarding the evaluation of the total questionnaire, 100% of the participating experts agreed or fully agreed with the questionnaire's relevance and scope.

In the third round, 9 of the original 14 specialists participated in the evaluation of the instrument. We asked experts if they agreed to the small changes made to the already satisfactory questions, and six (66.7%) agreed to all the amendments.

We also evaluated the reformulations of three questions. Only one question obtained indexes lower than 0.8 for clarity in the statement, clarity in the alternatives, and coherence between statement and answer. By presenting such a result, we removed this question from the final version of the instrument.

The final instrument consisted of 45 questions, all with satisfactory CVIs, with the lowest observed value of 0.8 (80%).

The mean CVI for the instrument is 0.954 (95.4%). Table 3 shows the CVIs obtained for each axis and the questionnaire as a whole.

**Table 3:** Analysis of Content Validity Index (CVI) for axes and the questionnaire as a whole obtained after the third round.

Axes		CVI %
Relevance		
	Questionnaire as a whole	100
	Axis 1	100
	Axis 2	92.9
	Axis 3	92.9
	Axis 4	100
	Axis 5	92.9
	Axis 6	100
	Axis 7	92.9
	Axis 8	100
	Axis 9	92.9
	Axis 10	100
	Axis 11	100
	Axis 12	100
	Axis 13	92.9
Scope		
	Questionnaire as a whole	100
	Axis 1	90
	Axis 2	80
	Axis 3	85.7
	Axis 4	92.9
	Axis 5	92.9
	Axis 6	100
	Axis 7	85.7
	Axis 8	100
	Axis 9	85.7
	Axis 10	100
	Axis 11	100
	Axis 12	100
	Axis 13	92.9

CVI: Content Validity Index. Measurement of the CVI in percentage for axes in the final instrument.

After we completed the third round, we evaluated the agreement between the judges using Gwet's AC2 Coefficient of Agreement^20^ with ordinal weights, given the nature of the response categories (totally disagree, disagree, agree, and totally agree) and the concentration of answers were in the two highest categories (I totally agree and agree). The coefficients were accompanied by 95% confidence intervals and p-values for the equality hypothesis test to zero. The coefficients show good or excellent agreement according to the criterion proposed by Altman,^21^ with the lowest coefficient of 0.792, observed for the scope of the questionnaire as a whole and of the individual axes, and the higher coefficient of 0.926 observed for relevance and relevance of the questions. Table 4 demonstrates the agreement analysis.

**Table 4:** Agreement analysis.

Analysis per question and axis		95% confidence interval	p-value
Questions			
	Clarity of statement	0.92 (0.89–0.95)	<0.001
	Clarity of alternatives	0.87 (0.83–0.91)	<0.001
	Coherence	0.87 (0.83–0.92)	<0.001
	Relevance	0.93 (0.9–0.95)	<0.001
Axes			
	Relevance	0.89 (0.84–0.94)	<0.001
	Scope	0.79 (0.70–0.88)	<0.001
	Global analysis	0.89 (0.88–0.91)	<0.001

We submitted the final instrument to a Portuguese teacher for orthographic analysis.

## DISCUSSION

After the third round of the Delphi technique, we validated a total of 44 questions. As of the second round, all axes and the whole questionnaire had CVI higher than 0.8 (80%) in scope and relevance. (Thus, it was considered validated even before the completion of the study.) The mean CVI of the instrument was high 0.95 (95%), and the overall coefficient of agreement of 0.89 and p-value lower than 0.001.

The present study used psychometrics and CVIs to validate a questionnaire created to assess a resident's knowledge of developmental and behavioral disorders. We stipulated a CVI of 0.8 to validate questions, axes, and questionnaire as a whole. For this, it was necessary to conduct three rounds of the Delphi technique. Despite the stipulated cutoff point of 0.8, the CVI of 89% of the questions was higher than 0.9. The CVI regarding the questionnaire's relevance and axes was also higher than 0.9, and we had only four axes with CVI ranging from 0.8 to 0.9.

The coefficient of agreement was also high, reaching 0.89 in the overall analysis. The coefficient of agreement was lower for the scope of the axes and questionnaire, which can be explained by the immense amount of content existing in the field of development and behavior.

Initially, the minimum number of judges considered was 10 specialists. However, we were able to obtain the participation of 14 specialists for the first phase. Only 37% of 37 specialists accepted our invitation, justified by the amount of time required to evaluate the questionnaire. According to the literature,^23,24^ the greatest importance is not in the number of participating judges but quality and representativeness. We consider the sample of judges satisfactory both because they meet the inclusion criteria and because they come from different regions of the country.

Despite the widespread presence and overarching impacts of developmental and behavioral disorders, some reasons do justify the difficulties experienced by the general pediatricians diagnosing problems on the frontlines: first, general pediatricians lack the preparation to diagnose and monitor these children; second, there is a shortage of sufficient time for pediatric consultations, a lack of validated screening instruments in Brazil, and professionals lack the necessary training to use these instruments; finally, we emphasize the extremely small number of professionals, multidisciplinary teams, and development and behavior specialized services throughout the country. In addition to the issues mentioned above, there are no mandatory course-load minimums (minimum credit requirements) stipulated by the National Commission of Medical Residency in Brazil.^10^

When validated, we expect that the medical community would consider this questionnaire a reliable tool for evaluating residents’ knowledge on the subject and serve as a means of diagnosing possible training deficits of the pediatricians in question. This instrument will be applied to the evaluation of residents in a future study. The validated instrument can be found in the annexes.

The strengths of this study include the widespread geographical distribution of specialists and their representativeness, the higher-than-expected sample size, the validation of the questionnaire with high CVIs already shown in the second round of the Delphi technique, and, finally, the use of the coefficient of agreement as one of the reliability measures.

As for the study's limitations, we cite that the evaluation of this field of study through multiple choice questions, although practical, may not cover all the peculiarities and details. We must also mention the absence of a pilot test to evaluate other aspects of reliability and the power to discriminate questions.

Another limitation was the lack of interaction between the members of expert's panel. The dialogue is an important factor for reaching a consensus. However, operational limitations have made the process impracticable.

In conclusion, we constructed a questionnaire to assess knowledge in diagnosing developmental and behavioral disorders. With CVI greater than 0.8 and high agreement of the 14 specialist judges, we consider the 44 questions, all 13 axes, and the questionnaire as a whole validated. Thus, we consider the questionnaire sufficiently relevant and comprehensive to evaluate residents’ knowledge regarding the diagnosis of developmental and behavioral disorders.
